# Talking About Privilege: Framing Inequality as Advantage Is More Likely for Inequality in Positive Than in Negative Outcomes

**DOI:** 10.1177/01461672241265779

**Published:** 2024-08-06

**Authors:** Annette Malapally, Susanne Bruckmüller

**Affiliations:** 1 Friedrich-Alexander-Universität Erlangen-Nürnberg, Germany

**Keywords:** inequality, privilege, framing, valence, social media

## Abstract

Inequality is often one-sidedly framed as disadvantage, a practice identified as problematic by empirical research and critical scholarship, as it renders privilege invisible and shapes perceptions of and reactions to inequality. Importantly, inequality can mean differences in positive (e.g., promotions) or negative outcomes (e.g., harassment). Drawing on cognitive processes involved in (group) comparisons and the processing of positive and negative content, we predict that the valence of outcomes moderates the preference for disadvantage (vs. advantage) frames. We coded social media posts on gender inequality (Study 1, *n* = 1,402) and had participants in an online experiment (Study 2, *n* = 164) describe gender and sexual orientation inequality in positive and negative outcomes. Confirming hypotheses, people overall used disadvantage frames more, but were more likely to use advantage frames for inequality in positive (compared with negative) outcomes. We discuss theoretical implications for inequality framing research and practical implications for privilege awareness interventions.

Inequality is a “defining challenge of our time” (United Nations, n.d.). How people perceive and respond to inequality critically depends on how it is being described (e.g., Iyengar, 1990; Kraus et al., 2022). Among many possible ways to describe inequality, one consequential variation is between framing it as one group’s advantage (or privilege), such as saying that men earn more than women, or another group’s disadvantage, like saying that women earn less than men (e.g., Bruckmüller et al., 2017; Dietze & Craig, 2021). Research has documented a pervasive tendency to frame inequality as disadvantage rather than as advantage (e.g., Jun et al., 2022; Malapally et al., 2024; Phillips et al., 2022). Scholars and activists have long emphasized that this creates a problematic blindness to privilege and have advocated for a more holistic perspective (e.g., DiTomaso, 2015; McIntosh, 1988). Thus, the question when (and why) people frame inequality as disadvantage and when they may (also) use advantage frames is important for both theoretical and social justice considerations.

Importantly, inequality can mean systematic differences in various aspects of life, including positive outcomes, such as income, promotions, or opportunities, as well as negative outcomes, such as obstacles, harassment, or violence. Both can be framed as advantage or disadvantage. Disadvantage can mean having more of a negative outcome (e.g., experiencing more harassment), or less of a positive one (e.g., earning less). Privilege can mean being advantaged by having more of a positive outcome (e.g., earning more), or less of a negative one (e.g., less harassment, see Table 1). We propose the valence of outcomes as a key moderator of inequality framing that has largely been overlooked by previous research. Based on relevant social cognitive processes and associated linguistic practices, we predict that the overall preference for disadvantage frames is more pronounced for negative than for positive outcomes. Applied to the examples above, this would mean a strong tendency to frame unequal experiences of harassment (a negative outcome) as disadvantage (lower row of Table 1), but weaker preferences for framing income differences (a positive outcome, upper row). In the following, we first discuss previously documented habits of framing inequality (and exceptions from them), why they matter, and what processes have been suggested to explain these tendencies, before we outline why we expect the valence of outcomes to be critical.

**Table 1.** Examples of Inequality in Positive and Negative Outcomes Framed as Disadvantage and Advantage.

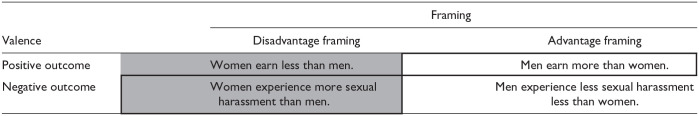

*Note.* H1 predicts an overall predominance of disadvantage framing (= cells shaded in gray) over advantage framing (not shaded). H2 predicts a moderation of framing by valence such that the combinations in the cells with double border are overall used more frequently than the combinations in the other two cells.

## Advantage and Disadvantage Framing Matter

Saying that one group is better off than another (advantage framing) or that the latter group is worse off than the former (disadvantage framing) may seem like a rather subtle, perhaps even trivial variation. Yet, it is one that can influence how people perceive, explain, and react to inequality. To give one empirical example, framing gender inequality as women’s disadvantage leads people to focus explanations and solutions for inequality more on women than a framing as men’s advantage (e.g., Bruckmüller & Braun, 2020). Other research has identified effects on physiological reactions, legitimacy appraisals, support for interventions, and more (e.g., Bruckmüller et al., 2017; Dietze & Craig, 2021; Dover, 2022).

A social cognitive explanation for such effects is that the target of a comparison (e.g., women in “women earn less than men”) becomes salient and is drawn on more in subsequent processing than the referent (here: men), while the referent becomes the less visible standard in relation to which the target is being judged. That is, hearing that women earn less than men or experience more sexual harassment than men tends to prompt questions tapping into what it is about women that causes their lower earnings or that makes them targets of harassment—implicitly measuring them against the unquestioned, “normal” standard of men’s earnings and relatively higher freedom from harassment (see Bruckmüller & Braun, 2020; Hegarty & Bruckmüller, 2013; Lowery et al., 2007). In line with this, critical scholars have long argued that chronic disadvantage framing implicitly stigmatizes disadvantaged groups and normalizes privilege—which ultimately helps uphold systems of inequality (McIntosh, 1988, 2012; Pratto & Stewart, 2012). In sum, it matters how inequality is framed, and persistently framing it as disadvantage is problematic.

## Inequality Is Often (But Not Always) Framed as Disadvantage

In public discourse, disadvantage frames are more common. Tweets about Black disadvantage are more common than tweets about White privilege (Malapally et al., 2024). U.S. news media use disadvantage more than advantage frames to describe gender and racial inequality (Jun et al., 2022). Experimental participants are more likely to frame these inequalities as disadvantage (Jun et al., 2022, U.S. samples) and more likely to focus on women than on men when explaining gender inequality (Braun et al., 2023; Bruckmüller & Braun, 2020, U.K. samples). Even scientific articles on inequality more often consider disadvantaging than advantaging mechanisms (Phillips et al., 2022).

### Why Is Disadvantage Framing More Common?

Previous research has often explained this with different group interests served by different frames. Being confronted with own privileges, especially if they appear unearned, can threaten the higher status and social identity of members of advantaged groups. Disadvantage framing helps deflect these threats by shifting the focus away from own advantages (Dover, 2022; Pratto & Stewart, 2012). Members of disadvantaged groups may also prefer disadvantage frames as they may be more conducive to drawing attention to their plight and raising sympathy from advantaged groups (see Harth et al., 2008; Lowery et al., 2012).

One can also approach the question of how people frame inequality from a norm theory–based perspective on how people perceive and explain intergroup differences (e.g., Hegarty & Pratto, 2001). In a nutshell, normative, high-status identities such as Whiteness and male gender tend to be taken as the default of personhood (e.g., Smith & Zárate, 1992), while lower status groups stand out as deviations from that norm (Hegarty & Pratto, 2001). Consequently, people tend to approach intergroup differences by comparing non-normative, lower status groups to normative, higher status groups (Hegarty & Bruckmüller, 2013). Applied to inequality framing, this translates into a focus on how outcomes of the disadvantaged (i.e., lower status) group differ from those of the advantaged group, that is, disadvantage framing (see also Lowery et al., 2007).

### When Inequality Is Framed as Advantage

A systematic exception has been observed for wealth inequality. Jun et al.’s (2022) participants more often framed wealth inequality as rich people’s advantage than as poor people’s disadvantage, and even more often with no focus on either group. One possible explanation for this may be differences in perceived legitimacy. Framing inequalities perceived as *illegitimate* (e.g., gender and racial inequality) as disadvantage allows advantaged groups to deflect status and identity threats (Phillips et al., 2022). Framing inequalities perceived as *legitimate* (e.g., wealth inequality) as advantage enables the advantaged to feel self-esteem and pride (Chow et al., 2008; Harth et al., 2008). The evidence for such a key role of legitimacy is somewhat mixed. Participants asked to describe racial and gender inequality from the perspective of someone who finds it illegitimate (vs. legitimate) used more disadvantage frames (Jun et al., 2022; Study 4). Yet, participants perceived wealth inequality as illegitimate overall (Jun et al., 2022; Study 3) and still used disadvantage frames least often to describe it. This suggests that legitimacy matters, but is not the only relevant factor here.

## Framing Inequality in Positive and Negative Outcomes

Specifically, we propose the valence of outcomes as another variable that critically shapes advantage and disadvantage framing. We deduce this from (social) cognitive principles and associated linguistic habits involved in comparative thinking and the processing of positive and negative content. Taken together, these processes make certain combinations of frame and valence more likely than others (see also Table 1). Importantly, we assume that these processes can operate in parallel and our hypotheses reflect the predictions that result when considering them together.

### Processing and Communicating Positive and Negative Content

The *correspondence principle* (Bouts et al., 1992; Woltin & Epstude, 2023) posits that outcomes are most readily attributed to causes that correspond to them in some important characteristic, for example, negative events tend to be attributed to negative (rather than positive) causes. Applying this principle more generally would mean that people more readily associate things that correspond in valence than those that do not. Importantly, the combinations of disadvantage framing and negative outcomes (e.g., “women experience more harassment than men”) and of advantage framing and positive outcomes (e.g., “men earn more than women”) correspond in valence, while advantage framing in negative outcomes (e.g., “men experience less harassment”) and disadvantage framing in positive outcomes (e.g., “women earn less”) do not. Because of this (non-)correspondence in valence, people may more readily connect disadvantage framing with negative outcomes and advantage framing with positive outcomes and therefore use these combinations more often in communication than the noncorresponding combinations.

Two further phenomena lead to the same prediction: first, the negativity bias, by which more attention and higher weight are given to negative information (e.g., Kahneman & Tversky, 1979; Rozin & Royzman, 2001). The highest negativity bias should be created by combining information about a negative outcome with a focus on the negative situation of being disadvantaged. Second, a positivity norm in communication leads people to focus on the positive and omit negative information (Bergsieker et al., 2012; Brown, 2015). The combination of advantage framing and positive outcomes fits this norm best, as it combines two positives.

In sum, combinations that correspond in valence (disadvantage framing and negative outcome, advantage framing and positive outcome) should overall be more common than noncorresponding combinations (disadvantage framing and positive outcome, advantage framing and negative outcome). This leads to the prediction of more disadvantage framing for negative outcomes and more advantage framing for positive outcomes.

### Processing and Communicating (Intergroup) Comparisons

In addition to the abovementioned tendency to compare non-normative with normative groups (Hegarty & Bruckmüller, 2013), people tend to focus on groups that show more of a certain behavior or have a certain attribute more. For example, Hegarty and Pratto’s (2001) participants focused their explanations of differences between gay and straight men, for example, in how much they followed a medical regimen, more on gay (non-normative group) than on straight men (normative group) and also more on whichever group was said to show more of the respective behavior. This finding fits with the more general observation that people tended to compare larger quantities (of a behavior) against smaller ones rather than vice versa (see also Matthews & Dylman, 2014), and the very basic perceptual principle that the presence of features is easier to perceive and draws more attention than their absence (*the feature-positive effect*, Newman et al., 1980; see also Meyvis & Yoon, 2021). Accordingly, people are more likely to frame differences in the form of “A more than B” rather than “B less than A,” and they also like “more than” comparisons better, agree with them more, and perceive them as more likely to be true (*the more-less asymmetry*, Hoorens & Bruckmüller, 2015; Skylark, 2021).

Taken together and applied to inequality framing, this means that thinking and speaking about the presence of an outcome, whether it is positive (e.g., earning more) or negative (e.g., experiencing more harassment), matches basic cognitive processes and associated linguistic habits better than thinking and speaking about their absence (i.e., earning less, experiencing less harassment). This again results in the prediction of more disadvantage framing for negative outcomes and more advantage framing for positive outcomes.

## Present Research and Hypotheses

### Hypotheses

In sum, several cognitive and communicative processes can be applied to inequality framing. First, given the overall predominance of disadvantage framing in previous research (e.g., Jun et al., 2022) and the sound theoretical basis for predicting it (e.g., Chow et al., 2008; Hegarty & Bruckmüller, 2013), we expect to replicate this overall tendency:

**Hypothesis 1 (H1):** People use more disadvantage than advantage frames to describe inequality.

Second, we predict the valence of outcomes to moderate this overall tendency such that advantage framing is relatively more common for positive (compared with negative) outcomes. This is because positive outcomes should be associated more with advantage than negative outcomes (correspondence principle), because the presence of positive outcomes should be easier to perceive than the absence of negative outcomes (feature-positive effect), and because there is a pervasive linguistic habit to prefer more-than over less-than comparisons (more-less asymmetry). Consequently, we predict that the overall tendency toward disadvantage framing (H1) will be attenuated for positive (compared with negative) outcomes:

**Hypothesis 2 (H2):** When people describe inequality in positive (negative) outcomes, they are more likely to use advantage (disadvantage) frames than when they describe inequality in negative (positive) outcomes.

Note that the processes predicting an overall tendency toward disadvantage framing (H1) and those predicting a moderation by valence (H2) are not mutually exclusive and can operate at the same time (just like Hegarty & Pratto, 2001, observed the joint occurrence of both, a tendency to focus on non-normative groups, and on groups that show more of a given behavior). Accordingly, we base our hypotheses on the confluence of these processes. Importantly, the main goal of the present research is to first establish whether valence of outcomes does indeed moderate inequality framing in predictable ways.

### Preliminary Evidence

Preliminary evidence for such a moderation comes from communication science. Gandy and colleagues (1997; Gandy & Li, 2005) analyzed journalistic articles on racial disparities in exposure to risks. They differentiated various kinds of frames, including *gain* and *loss frames*. They found a higher prevalence of *White gain framing* (e.g., “White people are more likely to survive.”) than of *White loss framing*, although “non-loss framing” may be more precise (“White people are less likely to die.”). In contrast, *Black loss framing* (“Black people are more likely to die”) was more frequent than *Black gain* (or more precisely, nongain) *framing* (e.g., “Black people are less likely to survive.”). This dovetails with our predictions. The more common White gain and Black loss frames relate closely to advantage frames for positive (survival) and disadvantage frames for negative (death) outcomes, while the cognitively more complex Black nongains (nonsurvival) and White nonlosses (nondeaths) overlap with disadvantage in positive, and advantage in negative outcomes (i.e., the two cells of Table 1 that we predict will be used less frequently).

Note, however, that Gandy and colleagues (1997, Gandy & Li, 2005) examined elite (media) frames while we investigate here how lay people use these frames. The present research thus translates their findings for gain and loss frames of relative risk into the terminology of psychological inequality framing research and builds on them to derive explicit predictions on the interaction of frame and valence. The present research is thus, to the best of our knowledge, the first to specifically test hypotheses on inequality framing in positive and negative outcomes (or gains and losses) by lay people and the first to do so experimentally (Study 2).

### Present Research: Overview

In Study 1, we manually coded social media posts on gender inequality to test the hypotheses in real communication (in English). We chose gender inequality because previous research in this context has documented a predominance of disadvantage framing, but with some variation (Jun et al., 2022), and because it allowed us to explore effects of own group membership (see below). Study 2 was a randomized online experiment, where participants formed sentences about gender and sexual orientation inequality in positive and negative outcomes. We added sexual orientation, because it is a basis of real-life inequalities (e.g., Bereswill & Ehlert, 2023) that has been largely ignored by previous research on inequality framing (see Hegarty et al., 2020, for a rare exception). Study 2 also extended the findings of Study 2 to a second language (German).

All analyses were run using R 4.3.0 (R Core Team, 2023). For specific packages used, see online supplement. We report all manipulations, measures, and exclusions. All significance tests are two-sided, with α = .05.

## Study 1

### Method

#### Design

In Study 1, we analyzed how women and men framed gender inequality in a manually coded corpus of social media posts from 2021. The posts (tweets) were collected from Twitter (now called X). The respective tweet IDs are available from the authors at request only, due to restrictions set by Twitter. They can be used to re-create the raw data set using the Twitter Application Programming Interface (API), unless a tweet has been deleted in the meantime.^1^ This study was not preregistered. Python and R scripts, coding scheme, and codebook can be found here: https://osf.io/b49nj/.

#### Data Collection

**Figure 1.**
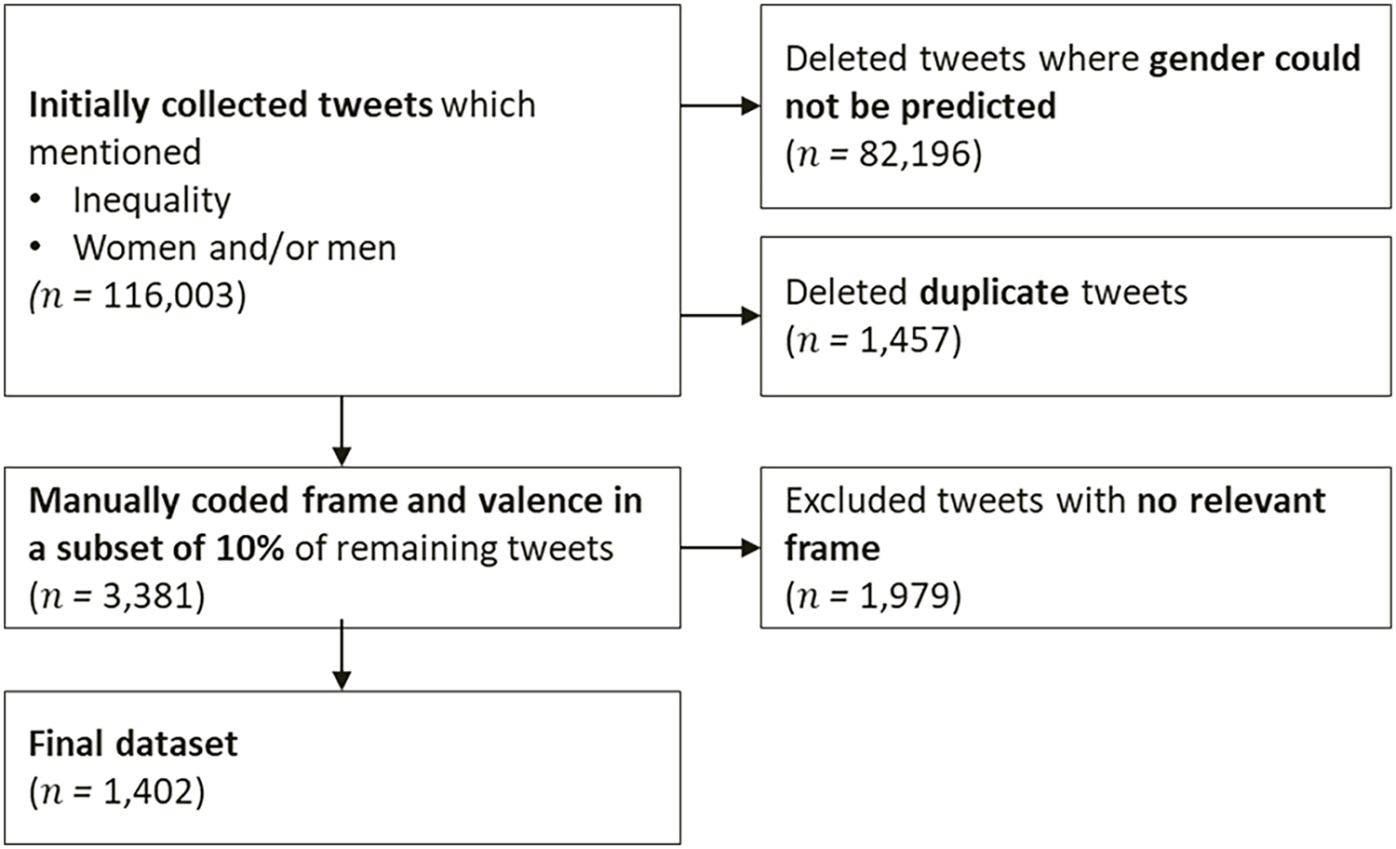
Data Collection and Filtering Process.

We queried and downloaded tweets through Twitter’s API, which allows developers and researchers to access information from Twitter. For every hour of each day in 2021, we downloaded up to 500 tweets, following Malapally et al. (2024).^2^ The query matched tweets that mentioned inequality between women and men, that is, included any of the keywords “inequality,” “unequal,” or “inequalities” and at least one keyword referring to women (“woman,” “women,” “girl,” or “female”) or men (“man,” “men,” “boy,” or “male”). API parameters were used to exclude retweets and non-English tweets. Downloads were completed in November 2022, so all tweets were at least 10 months old. This resulted in an initial data set of 116,003 tweets. Figure 1 illustrates the full data collection and filtering process.

#### Data Preparation

##### Preprocessing

To predict the gender of the users who had posted the tweets, we used the *gender-guesser* library (Pérez, 2016) that classifies names as (mostly) male, (mostly) female, or ambiguous (“androgynous”). We retained only tweets whose author’s first name (as given in the username) was predicted to be (mostly) male or female. Duplicate tweets were deleted. From the remaining tweets, we drew a random subsample of 10% for manual coding, resulting in a data set of 3,381 tweets.

##### Manual Coding

We manually coded tweets for framing and valence of outcomes (see Tables 2 and 3 for examples). Framing was coded as describing gender inequality with a focus on women (i.e., a disadvantage frame), on men (advantage frame), with no particular focus, or as other (i.e., tweets that did not describe inequality between women and men or that described [alleged] advantages of women or disadvantages of men). Regarding valence, tweets were coded as mentioning outcomes that were positive, negative, both positive and negative, abstract, or other (i.e., tweets that described none of these outcomes or where frame was “other”). Two independent raters coded 200 tweets, with substantial interrater reliability, κ_focus_ = .85, κ_valence_ = .83. One rater coded the remaining tweets. Tweets with no relevant frame were excluded, leaving a final data set of 1,402 tweets.

**Table 2.** Example Tweets for Manual Coding of Frame in Study 1.

Label	Example
Focus on women	. . . FGM is An extreme form of **Discrimination Against women**, Reflecting deep-rooted gender Inequality. . . .
Focus on men	**Men still speak and participate more** — demonstrating that the “chilly climate” of college classrooms still matters for gender inequality in education. . . .
No focus	. . . If a system produces **disparate outcomes for men and for women**, we’d say it’s sexist. . . .
Other	. . . The growth of inequality in US has been extraordinary in last 40 years. If income equality was same now as in 70s, men on $36,000 a year now would be on $65,000. . . . . . . Men have shorter lives, particularly in deprived countries and areas. . . . My daughter, a Major in the US Army, has equal rights. She can fight, shoot, . . . go to war, and die just as “equal” as any man. . . . Come on man, there is no inequality.

*Note.* Sections of the tweets’ text that are relevant for coding the respective label are in bold.

**Table 3:** Example Tweets for Manual Coding of Valence in Study 1

Label	Example
Positive	. . . unequal treatment—the men have more **services & support** . . .
Negative	. . . The pandemic has aggravated gender inequality, increased **informal work**, and **difficulty in accessing health care** . . .
Both	. . . It does nothing to end **violence** against women or gender inequality. And guess what? At the end of today, women will still **get paid** 35% less than men . . .
Abstract	It is timely and critical to address **gender #stereotypes** in our societies that are **perpetuating gender inequalities** . . .

*Note.* Sections of the tweets’ text that are relevant for coding the respective label are in bold.

Coding the excluded tweets in more detail (see online supplement for coding scheme and detailed analysis), we found a small number of tweets about reverse discrimination (e.g., “millennial women earn more than men the same age”; *n* = 56) and about interventions against inequality (e.g., “if you really want to help . . ., look a policies that . . . empower women”; *n* = 134). The remaining excluded tweets were “noise,” that is, not about gender inequality (e.g., “Black women are . . . more likely to die in childbirth than white women,” which covers racial rather than gender inequality; “I read concentration inequality and I retweet,” which does not describe a specific inequality).

### Results

#### Descriptive Results

Tweets were predominantly posted by women (63.6%). Most tweets used a disadvantage frame (66.0%), followed by those with no focus (29.3%), and those using an advantage frame (4.6%). Most tweets described inequality in abstract terms (45.6%), followed by negative outcomes (24.7%), positive outcomes (20.8%), or both (9.0%). See Table 4 for frequency of frames by valence overall and by gender of users.

**Table 4.** Frequency of Frames by Valence Overall and by Gender of Users.

Gender of users	Valence	Disadvantage frame	Advantage frame	No focus
Overall	Neg.	309 (37.1%)	5 (7.8%)	32 (8.5%)
	Pos.	189 (22.7%)	44 (68.8%)	58 (15.3%)
	Abs.	336 (40.3%)	15 (23.4%)	288 (76.2%)
Women	Neg.	214 (39.4%)	2 (4.9%)	23 (10.7%)
	Pos.	115 (21.2%)	31 (75.6%)	24 (26.8%)
	Abs.	214 (39.4%)	8 (19.5%)	167 (78.0%)
Men	Neg.	95 (32.6%)	3 (13.0%)	9 (5.5%)
	Pos.	74 (25.4%)	13 (56.5%)	34 (20.7%)
	Abs.	122 (41.9%)	7 (30.4%)	121 (73.9%)

*Note.* Neg. = negative valence, pos. = positive valence, abs. = abstract.

#### Hypotheses Testing

**H1:** We recoded framing as binary (advantage, disadvantage), that is, excluded tweets coded as “no focus” or “other.” As predicted, disadvantage framing was more common (93.4%) than advantage framing (6.6%). A chi-square-goodness-of-fit test confirmed that these proportions were not equal, χ^2^(1, *n* = 991) = 748.05, *p* < .001, *V* = 0.87, 95% CI_*V*_ = [0.71, 0.93].
**H2:** We recoded valence as binary (positive, negative), by excluding tweets describing both or abstract outcomes. Supporting H2, participants used advantage frames more for positive (89.8%), compared with negative outcomes (10.2%), and used disadvantage frames more for negative (62.0%), compared with positive outcomes (38.0%). This frame by valence interaction was significant, χ^2^(1, *n* = 547) = 49.04, *p* < .001, *V* = 0.30, 95% CI_*V*_ = [0.21, 0.38]. Comparing frame frequencies per valence showed that the preference for disadvantage framing, although more pronounced for negative outcomes (98.4%), persisted for positive outcomes (81.1%).^3^

#### Exploratory Analyses

##### Including Other Inequality Frames

We also inspected the frame by valence interaction for tweets with no focus (i.e., no clear advantage or disadvantage frame) and for tweets about abstract outcomes (not codable as positive or negative).^4^ It was significant, χ^2^(4, *n* = 1276) = 232.61, *p* < .001, *V* = 0.30, 95% CI_*V*_ = [0.26, 0.34]. Participants used advantage frames most for positive outcomes (68.8%), followed by abstract descriptions (23.4%) and negative outcomes (7.8%). They used disadvantage frames equally often for abstract differences (40.3%) and negative outcomes (37.1%), and less for positive outcomes (22.7%). They used no focus frames most for abstract differences (76.2%), followed by positive outcomes (15.3%) and negative outcomes (8.5%). All pairwise comparisons except the one between abstract and negative outcomes for disadvantage frames were significant (see supplement for full results).

##### Interaction of Frame, Valence, and Gender

We used a log-linear model to test whether frame and valence differed between tweets by women versus men. Tweets about both negative and positive outcomes were excluded because of low cell count. The final model retained main effects and two-way interactions, with a likelihood ratio of χ^2^(6) = 14.39, *p* = .026. Including the three-way interaction of frame, valence, and gender did not significantly improve model fit, χ^2^(4) = 8.81, *p* = .066, indicating similar results for women and men. Partial associations of the model terms showed that the two-way interaction of valence and gender was significant, χ^2^(2) = 6.89, *p* = .032, but the interaction of frame and gender was not, χ^2^(2) = 5.58, *p* = .062. Separate chi-square tests showed that men significantly more often tweeted about positive outcomes (23.7% of all statements posted by men) than women did (19.1%), χ^2^(1) = 4.30, *p* = .038, *V* = 0.05, 95% CI_*V*_ = [0.00, 0.10]; women tweeted more often about negative outcomes (26.8%) than men did (21.0%), χ^2^(1) = 5.90, *p* = .015, *V* = 0.06, 95% CI_*V*_ = [0.00, 0.11]. Men also described inequality more often in abstract terms (49.0%) compared with women (43.6%), but nonsignificantly so, χ^2^(1) = 3.83, *p* = .050, *V* = 0.04, 95% CI_*V*_ = [0.00, 0.10].

In sum, women and men framed inequality similarly and in line with hypotheses. However, compared with women, men more often mentioned inequality in positive and less often in negative outcomes.

### Discussion

As predicted, disadvantage frames were more common than advantage frames, but tweets about positive outcomes more often contained advantage frames than those about negative outcomes did. Notably, tweets on gender inequality were predominantly posted by women—although, globally, most Twitter users are men (Statista, 2022). This supports earlier research that social media discourse on inequality often emanates from disadvantaged groups (Freelon et al., 2018). However, we found no effect of group membership on frame use. This stands in contrast to earlier experiments where members of privileged groups, but not disadvantaged groups, avoided using advantage frames to describe illegitimate, compared with legitimate inequality—arguably, to deflect identity threats (Dover, 2022). One explanation could be that in contrast to experimental participants, Twitter users speak about inequality on their own accord. Men who decide to tweet about gender inequality may more readily acknowledge their advantages (cf. Dover, 2022). For some people, acknowledging privilege can bolster self-regard, for example, those with a liberal political orientation (Phillips & Lowery, 2020).

However, compared with women, men more often tweeted about inequality in positive and less often in negative outcomes. One explanation may be that people use their own social identity as an organizing framework, or “lens,” through which they perceive their social world. This lens-based account of social perception (Petsko et al., 2022) was developed for the context of stereotyping in person perception, but it may also apply to the perception of social phenomena like inequality. Individuals may use their own group identity as a lens through which they see inequality between their own and other groups. As a result, they sharpen their focus on what they experience or see their group experience more in everyday life. The disadvantaged group (here: women) would therefore focus (even more) on negative outcomes; the advantaged group (here: men) would, at least relatively speaking, focus more on positive outcomes.

To replicate the findings of Study 1 in a controlled setting, and thus to enhance internal validity, we conducted a preregistered experiment (Study 2). This also served to extend the results to a second language (German) and to a second inequality domain (sexual orientation). Finally, extending earlier research (Dover, 2022; Jun et al., 2022), a controlled experiment also allowed us to measure perceived legitimacy and ingroup identification as potential moderators of frame use.

## Study 2

### Method

#### Design

Study 2 had a 2 (domain: gender, sexual orientation) × 2 (valence of outcomes: positive, negative) within-subjects design. Study design, planned sample size, exclusion criteria, hypotheses, and procedures for calculating dependent variables, hypotheses testing, and follow-up analyses were preregistered (https://aspredicted.org/H4N_SMV) and are all reported.^5^ Materials (original German and translated English version), scripts, data, and codebook can be found here: https://osf.io/b49nj/.

#### Participants

An a priori power analysis for a 2 (domain: gender, sexual orientation) × 2 (valence of outcome: positive, negative) × 2 (framing: disadvantage, advantage) repeated measures analysis of variance (ANOVA) resulted in a required sample size of 160 to detect an effect of *f* = 0.10, with α = .05, 1 – β = .80, and *r* = .60. We recruited 178 participants to account for drop-out (exceeding the preregistered sample size of *n* = 172). A flyer with the participation link was distributed in local social media groups and in public locations in a German city.

We excluded participants who did not have at least good knowledge of German (*n* = 12), or who formed less than nine (out of 12) critical sentences (*n* = 10). No participant indicated to not have answered the questionnaire seriously. This left 164 participants for hypotheses testing, meeting the required sample size. This included 95 women, 63 men, and six nonbinary persons as well as 133 straight, five gay (of any gender), and 23 other queer persons. Three participants did not report sexual orientation. Participants with more than one missing value in any of the exploratory composite variables (*n* = 14) were treated as missing and excluded from all exploratory analyses.

#### Procedure and Materials

##### Manipulation of Inequality Domain and Valence

After an example using an unrelated topic, participants formed 14 sentences from prompts. Of the 12 critical sets of prompts, half were about gender and half about sexual orientation–based inequality. Of these, three sets each were about positive and negative outcomes. An example set for gender inequality in positive outcomes is “women,” “men,” and “leadership positions,” and for sexual orientation inequality in negative outcomes “gay people,” “straight people,” and “victims of violence.” The order in which the disadvantaged and advantaged group were mentioned was randomized. In addition, there were two sets of distractor prompts (e.g., “different sexual orientations,” “sex education in school”).

##### Measure of Framing

The sentences written by participants (*n* = 2,048) were coded for frame and content. Sentences were coded as focusing on the advantaged group (i.e., men; straight people), on the disadvantaged group (i.e., women; gay people), having no focus on either group, or as other (i.e., nonsense answers or answers that mentioned neither group). Regarding content, sentences were coded as describing gender inequality, denying inequality, describing “reversed” inequality (i.e., describing an alleged disadvantage of the advantaged group or an advantage of the disadvantaged group), calling for change, assigning blame, or as other (e.g., nonsense answers, “no idea”). Two independent raters coded around 10% of sentences (*n* = 240) and achieved good reliability for frame, κ_frame_ = .79, and content, κ_content_ = .84. One rater coded the remaining sentences alone. Table 5 shows example sentences for the coding of frame (see online supplement for content coding examples).

**Table 5.** Example Sentences for Manual Coding of Frame in Study 2.

Label	Example
Focus on the advantaged group	“**Men** are more likely to get a raise after spending an equal amount of time in a company.”
Focus on the disadvantaged group	“**Gay people** more often become victims of violence than straight people.”
No focus	“More and more **women** are active in politics, although **men** are still the majority.”
Other	“I don’t have any experience with this.”

*Note.* Sections of the text that are relevant for coding the respective label are in bold.

For the main dependent variables, namely, the proportions of (dis)advantage frames used by participants, we considered all sentences focusing on the disadvantaged group and describing inequality as containing a disadvantage frame, and all sentences focusing on the advantaged group and describing inequality as containing an advantage frame. Sentences describing inequality with no focus on either group were coded as no focus frame. Sentences with no focus (*M* = 0.07, *SD* = 0.15) and sentences in the remaining content categories (*M* = 0.16, *SD* = 0.20) were quite rare, so we did not analyze them further. We then computed eight variables representing the number of advantage and of disadvantage frames in each of the four sentences categories, that is, gender inequality in positive and in negative outcomes as well as sexual orientation inequality in positive and negative outcomes (all relative to the respective participant’s total number of sentences in the respective category coded as describing inequality).

##### Perceived Inequality

Next, we measured the perceived extent of gender and sexual orientation inequality with one item each (“Men and women are now equal in our society.,” “Gay people are now treated just like straight people.”) on a 7-point Likert-type scale, as were all following items (unless noted otherwise).

##### Perceived Legitimacy

We measured the perceived legitimacy of gender (Cronbach’s α = .64) and sexual orientation inequality (Cronbach’s α = .63) with four items each (e.g., “Social inequality between men and women is unfair,” “I think it is fair that some people have more difficulties in some areas because of their sexual orientation.”).

##### Political Orientation

Political orientation was measured on two bipolar items from “strongly left”/“strongly progressive” to “strongly right”/“strongly conservative” (*r* = .74).

##### Group Membership

Participants indicated their gender and sexual orientation in two open responses and one rater coded responses as membership in the disadvantaged group (i.e., women, gay people), the advantaged group (i.e., men, straight people), or as another identity (e.g., nonbinary people, bisexual people).

##### Ingroup Identification

Identification with the gender (Cronbach’s α = .68) and sexual orientation ingroup (Cronbach’s α = .72) was measured with four items each (e.g., “Being [a woman] has little to do with how I think about myself.” [reverse coded]; “Being [gay] is an important aspect of who I am.”; adapted from Wilkins et al., 2017, by inserting each participant’s previous group membership responses in the brackets).

##### Demographic Data

We also measured participants’ age, highest education, current occupation, and knowledge of German.

##### Debriefing

Finally, participants could leave comments in open text boxes, were debriefed, and thanked. As compensation, they had the option to enter a raffle for 2 × 25€.

### Results

#### Hypotheses Testing

**Figure 2.**
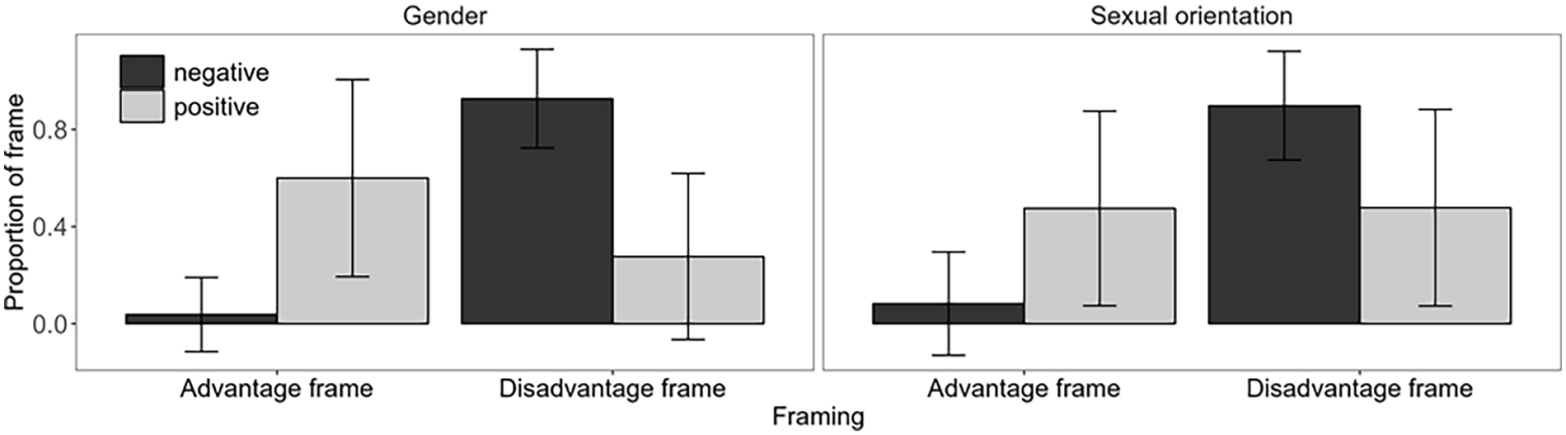
Proportions of Disadvantage and Advantage Frames for Negative and Positive Outcomes, in the Gender and Sexual Orientation Domain, in Study 2 *Note.* Error bars show the standard deviation per experimental group.

We conducted a 2 (domain: gender, sexual orientation) × 2 (framing: disadvantage, advantage) × 2 (valence of outcome: positive, negative) repeated measures ANOVA, including main effects and all interactions to test H1 and H2. The dependent variables were the proportions of each frame per condition (see Figure 2; see Table 6 for proportion of frames on the level of sentences). We used repeated measures ANOVA because domain and valence were manipulated within-participants and both kinds of framing were measured for each participant:

**H1:** As predicted, participants overall used more disadvantage (*M* = 0.65, *SD* = 0.41) than advantage frames (*M* = 0.30, *SD* = 0.40). The main effect of framing was significant, *F*(1, 123) = 133.29, *p* < .001, $\eta_{p}^{2}$ = .52, 95% confidence interval (CI) $\eta_{p}^{2}$ = [.40, .61].
**H2:** The predicted framing by valence interaction was significant, *F*(1, 123) = 322.66, *p* < .001, $\eta_{p}^{2}$ = .72, 95% CI $\eta_{p}^{2}$ = [.64, .78]. Participants more often used advantage frames for positive outcomes (*M* = 0.54, *SD* = 0.41) than for negative outcomes (*M* = 0.06, *SD* = 0.19), *t*(272) = −17.6, *p* < .001, *d* = −1.06, 95% CI_*d*_ = [−1.24, –0.92]. They used disadvantage frames more for negative (*M* = 0.91, *SD* = 0.21) compared with positive outcomes (*M* = 0.37, *SD* = 0.39), *t*(271) = 19.6, *p* < .001, *d* = 1.18, 95% CI_*d*_ = [0.99, 1.41].

**Table 6:** Proportion of Disadvantage and Advantage Frames per Set of Prompts

Domain	Valence	Outcomes	*n*	Disadvantage frame	Advantage frame	No focus
Gender	Positive	Salary raises	118	24.6%	56.8%	18.6%
		Representation in politics	102	30.4%	59.8%	9.8%
		Leadership positions	116	25.9%	69.0%	5.2%
	Negative	Domestic violence	120	90.7%	2.3%	7.0%
		Sexual harassment	134	89.6%	6.7%	3.7%
		Poverty in old age	118	98.3%	1.7%	0%
Sexual orientation	Positive	Representation in the media	99	50.5%	44.4%	5.1%
		Representation in parliament	89	40.5%	53.9%	5.6%
		Chances to adopt a child	107	51.4%	45.8%	2.8%
	Negative	Experiences of violence	113	94.7%	5.3%	0%
		Experiences of discrimination	130	81.5%	13.9%	4.6%
		Psychological strain	82	97.7%	1.1%	1.1%

We also tested the other two possible post hoc comparisons (preregistered exploratory analyses). Participants used significantly more disadvantage than advantage frames for negative outcomes, *t*(296) = −38.34, *p* < .001, *d* = −2.22, 95% CI_*d*_ = [−2.96, –1.79], and more advantage than disadvantage frames for positive outcomes, *t*(286) = 3.78, *p* < .001, *d* = 0.22, 95% CI_*d*_ = [0.11, 0.35].

As the three-way interaction of frame, valence, and domain was significant, *F*(1, 123) = 20.88, *p* < .001, $\eta_{p}^{2}$ = .15, 95% CI $\eta_{p}^{2}$ = [.05, .26], we ran separate repeated measures ANOVAs for the gender and sexual orientation domain. Again, in line with H1, there was a main effect of framing for both domains — gender: *F*(1, 142) = 81.21, *p* < .001, $\eta_{p}^{2}$ = .36, 95% CI $\eta_{p}^{2}$ = [.24, .47], and sexual orientation: *F*(1, 127) = 107.28, *p* < .001, $\eta_{p}^{2}$ = .46, 95% CI $\eta_{p}^{2}$ = [.33, .55]. Disadvantage frames were used more often than advantage frames (gender: *M*s = 0.60 and 0.32, *SD*s = 0.43 and 0.42; sexual orientation: *M*s = 0.70 and 0.27, *SD*s = 0.39 and 0.37, respectively). Again, in line with H2, framing and valence interacted for both domains. However, the interaction was stronger for gender, *F*(1, 142) = 328.57, *p* < .001, $\eta_{p}^{2}$ = .70, 95% CI $\eta_{p}^{2}$ = [.62, .75], than for sexual orientation, *F*(1, 127) = 106.78, *p* < .001, $\eta_{p}^{2}$ = .46, 95% CI $\eta_{p}^{2}$ = [.33, .55] (see also Figure 2). In the supplement, we report the respective Bonferroni-corrected post hoc tests.^6^

In sum, for both domains, participants overall used more disadvantage than advantage frames, but used advantage frames more for positive than negative outcomes, supporting H1 and H2. Interestingly, inequality in negative outcomes was almost always framed as disadvantage, while inequality in positive outcomes was framed in a more balanced way.

#### Exploratory Analyses

##### Perceived Legitimacy

We tested perceived legitimacy, valence, and framing type as predictors of frame use in linear regressions with random intercepts for participants, separately per inequality domain. Perceived legitimacy did not predict frame use in either domain, gender: *b* = 0.01, *SE* = 0.02, 95% CI_*b*_ = [−0.04, 0.05], sexual orientation: *b* = 0.02, *SE* = 0.04, 95% CI_*b*_ = [−0.06, 0.09], and did not interact with valence and frame type, gender: *b* = 0.02, *SE* = 0.04, 95% CI_*b*_ = [−0.06, 0.11], sexual orientation: *b* = 0.01, *SE* = 0.08, 95% CI_*b*_ = [−0.15, 0.15]. See supplement for complete results of these regressions, and regressions excluding outliers.

##### Ingroup Identification and Group Membership

Ingroup identification (across all genders and sexual orientations) did not predict frame use, and group membership did not predict frame use for gender inequality. See supplement for full results of these analyses, and those excluding outliers. There were not enough gay participants to test group membership as a predictor for sexual orientation inequality frames. Excluding queer participants did not change the pattern of results for ingroup identification.

### Discussion

In line with hypotheses and with Study 1, disadvantage frames were, overall, more common than advantage frames, but considering inequality in positive outcomes increased the use of advantage frames. For gender inequality in positive outcomes, participants even used more advantage than disadvantage frames. This differs from the sexual orientation domain, where the respective frames were more balanced, and from Study 1, where even tweets about positive outcomes were mostly framed as disadvantage. Importantly, despite the predicted overall preference for disadvantage framing, a reversal for positive outcomes in some, but not all, cases is well in line with our moderation hypothesis and the underlying theoretical considerations. Arguably, different processes contribute to framing choices and their relative importance may vary with context. For example, the cognitive processes predicting a tendency toward advantage framing for positive outcomes (e.g., more-less asymmetry) may matter more in an anonymous online study, while the identity threat processes predicting a general preference for disadvantage framing (at least for inequalities perceived as illegitimate) are arguably more relevant for public social media posts.

Another notable difference to Study 1 is that while in Study 1, most participants were members of the disadvantaged group (women), in Study 2, most were members of the advantaged group (straight people). However, this is unlikely to have biased the present results. For gender inequality, group membership did not influence frame use. In addition, Study 2 had a substantial number of nonstraight participants and removing their data did not change results. We would, therefore, expect similar results for gay and straight participants, although empirically, this remains a question for future research.

Finally, perceived legitimacy of inequality did not moderate frame use. This stands in contrast with earlier research where perceived legitimacy predicted more use of advantage frames (Dover, 2022) and perceived legitimacy mediated the effect of inequality domain on the use of advantage and disadvantage frames (Jun et al., 2022; Study 3). However, previous studies are also partly inconsistent regarding effects of legitimacy on frame use. While perceived legitimacy affected disadvantage frame use, there was no effect on advantage frames (see Jun et al., 2022, supplementary analysis to Study 4). Moreover, the inconsistencies between our findings and earlier studies may partly be due to additional moderators, such as membership in the (dis)advantaged group (see above, even though neither group membership nor identification predicted frame use here) or the cultural context (the United States vs. Germany). More important for the present research question, Study 2 shows that considering inequality in positive outcomes can induce a perspective of advantage—independently of a communicator’s perceived legitimacy and their group membership.

## General Discussion

In line with hypotheses, disadvantage frames of gender and sexual orientation inequality were more common than advantage frames, and describing inequality in positive, compared with negative, outcomes made it more likely that people used advantage frames. The strength of this interaction varied between studies and inequality domains, but unequivocally, advantage (or privilege) frames were used more often to describe inequality in positive than in negative outcomes.

### Theoretical Implications

The overall preference for disadvantage framing of gender inequality replicates previous research (e.g., Jun et al., 2022) and extends it to the domain of sexual orientation. It also aligns with previous research on intergroup comparisons (Hegarty & Bruckmüller, 2013) and extends it to intergroup inequality. Group membership did not significantly affect frame use, which fits with previous theorizing that disadvantage framing serves the immediate interests of both advantaged and disadvantaged groups (e.g., Dover, 2022; Lowery et al., 2012), even though it may only benefit privileged groups in the long run by normalizing their privilege (McIntosh, 1988, 2012; Pratto & Stewart, 2012). The moderation by valence may also (partially) explain previously observed framing differences between different inequality domains (e.g., Jun et al., 2022). To the extent that wealth inequality is more often conceptualized in terms of positive outcomes (income, wealth) than gender and racial inequality, one would expect more advantage frames in this domain. Disentangling to what extent the valence of outcomes (versus other factors such as legitimacy) contributes to different framing preferences for different inequality domains is a task for future research.

The moderation by valence of outcomes also matches general principles like the feature positive-effect (Newman et al., 1980) and the more-less asymmetry (e.g., Hoorens & Bruckmüller, 2015) and extends them to inequality framing. It also aligns with the correspondence principle (Woltin & Epstude, 2023) and suggests it not only holds for causes and effects of similar valence but also for the association of advantage with positive outcomes and disadvantage with negative outcomes. Also in line with this principle is our finding that no focus frames were most often used to describe abstract differences between women and men (Study 1). A no focus frame neither presents inequality as advantage nor as disadvantage and may thus correspond less to specific (positive or negative) outcomes and instead to more abstract differences.

Finally, our findings are compatible with the *inequality framing model*, which holds that disadvantage and advantage framing influence perceivers’ reactions to inequality because they suggest different equity standards (Chow et al., 2010), that is, (dis)advantage frames suggest that a group has (less) more than would be fair. Disadvantage frames, therefore, suggest providing positive outcomes to or taking away negative outcomes from disadvantaged groups to achieve equity. In contrast, advantage frames suggest withholding positive outcomes from or giving negative outcomes to advantaged groups. As people are generally guided by a do-no-harm principle (Leliveld et al., 2009), they may find it less appropriate to frame inequality in terms of advantage than as disadvantage, particularly in negative outcomes—as it may put forward the idea of harming the advantaged group to achieve equity. In line with this, advantage in negative outcomes was the least used frame across our studies.

### Practical Implications

Advantage versus disadvantage framing is a relatively subtle variation because of the logical equivalence of the two variants. Nevertheless, it can affect how people perceive and react to inequality (Phillips et al., 2022), and because of this subtlety, its influence may often go unnoticed. This makes advantage and disadvantage framing a rather powerful linguistic tool.

From an applied perspective, it may be tempting to ask which of the two framings may be more conducive to challenging (or maintaining) inequality, and as we started this article by highlighting a one-sided perspective on disadvantage as problematic (DiTomaso, 2015; McIntosh, 1988; Pratto & Stewart, 2012), one may assume that we advocate for advantage framing. However, a predominant, one-sided advantage framing would be just as problematic. While disadvantage framing singles out disadvantaged groups and renders privilege invisible, empirically, it also leads to an easier recognition of inequality as problematic (Bruckmüller et al., 2017; Phillips & Jun, 2022) and elicits more sympathy and solidarity from advantaged groups (Harth et al., 2008; Lowery et al., 2012). Advantage framing can make otherwise inaccessible explanations and solutions of inequality visible (Bruckmüller & Braun, 2020), but can also cause reactance from members of advantaged groups (Phillips & Lowery, 2020). In short, advantage and disadvantage framing have a range of different consequences, defying a simple answer to the rather simplistic question of which framing is best (see Bruckmüller, in press).

Given that advantaging and disadvantaging mechanisms work together to create and maintain inequality (e.g., DiTomaso, 2015), it is important to make all of these mechanisms and their consequences visible—and currently, all too often, advantage remains invisible, even though favoritism toward dominant groups (and the absence of such favoritism toward subordinate groups) is a crucial inequality upholding mechanism (Brewer, 1999). Therefore, from a practical point of view, the most pressing question is how one can facilitate the (additional) use of advantage frames, to avoid one-sided views and to create privilege awareness among dominant groups, who have the power to change outcomes (Rueschemeyer, 2004).

Although of course awaiting more direct empirical tests, based on the present findings, we would predict that considering inequality in positive outcomes makes it easier to notice advantages and to start conversations about privilege—at least relative to a focus on negative outcomes. Practically, this would mean starting privilege awareness exercises with advantages in positive outcomes, to ease people into discussing and reflecting on privilege. Of course, discourse about inequality should not *only* focus on advantage, as considering both advantage and disadvantage, in positive and negative outcomes, is necessary to fully understand and address inequality (DiTomaso, 2015). However, privilege awareness can threaten advantaged groups (Dover, 2022; Lowery et al., 2007) and lead to reactance (Phillips & Lowery, 2020). Yet, it is also a privilege to be able to reject discrimination against disadvantaged groups while remaining oblivious to favoritism toward one’s own group (DiTomaso, 2015). One way to manage defensiveness is to make advantaged groups feel safe before addressing privilege, for example, via self-affirmation (Lowery et al., 2007). An additional strategy may be to start with positive outcomes to match cognitive and linguistic defaults best.

Leading by example, the well-known “invisible knapsack” of White privilege (McIntosh, 1988) describes 12 instances of privilege in positive outcomes (e.g., “neighbors will be . . . pleasant to me”), 12 in negative outcomes (e.g., “not to be . . . harassed by store detectives”), and two in both (e.g., “remain oblivious of the language and customs of persons of color, . . . without feeling . . . any penalty”). However, balanced valence has presumably rarely been a concern when designing privilege checklists and many consider mostly negative outcomes — for example, the Male Privilege Checklist (24 negative, for example, “not taught to fear walking alone after dark”; 14 positive, for example, “odds of being hired for a job”; two both; Deutsch, 2006), Heterosexual Privilege Checklist (22 negative, 16 positive, two both; Killerman, 2012), or Monogamous Privilege Checklist (42 negative, 20 positive, four both; Davis, 2011).

### Limitation and Directions for Future Research

In contrast to earlier research (Dover, 2022; Jun et al., 2022), our exploratory analyses showed no effect of perceived legitimacy (Study 2) or group membership (Studies 1 and 2) on frame use. As Jun et al. (2022) also argue, follow-up research should investigate which other factors impact the use of advantage versus disadvantage frames to dissolve these inconsistencies. The present research identifies valence of outcomes as one such moderator. Future research should determine whether valence of outcomes could be an alternative explanation for earlier findings that some inequalities (e.g., wealth inequality) are more often framed in terms of advantage than others (e.g., gender inequality; Jun et al., 2022) or whether both processes work in parallel. Such research would of course require varying both valence and legitimacy as experimental factors. As our main goal here was to first establish valence of outcomes as an important moderator, systematically testing its importance vis-à-vis other factors was beyond the scope of the present studies.

In Study 2, the experimental manipulation gave participants little leeway in describing inequality. This ensured internal, but limited external validity as it restricted them to mostly use disadvantage or advantage frames. Balancing this, Study 1 measured the use of no focus frames and found that they were most often used for abstract differences. Future research should experimentally replicate these findings as the no focus frame is a prevalent framing of some inequalities, for example, wealth inequality (Jun et al., 2022). The experimental manipulation of valence in Study 2 also restricted participants to describing the stipulated positive or negative outcomes. Critically, these prompts not only varied in their valence but also in their content (e.g., salary raise vs. harassment). Future research should replicate this for equivalent frames of positive versus negative outcomes, for example, being granted versus refused a salary raise (cf. Gandy et al., 1997). However, supporting the validity of our findings, Study 1 found the same interaction of frame and valence in spontaneous communication, that is, without presetting the outcomes participants wrote about. The combination of both studies thus provides robust support for our hypotheses.

The generalizability of the present findings is, however, restricted to the investigated languages and inequality domains. Regarding language, the few empirical studies on inequality frame use in real communication were conducted in English (Jun et al., 2022; Malapally et al., 2024). The results of Study 2 extend this to German, and align with findings in English as disadvantage frames were more frequent than advantage frames for gender (and sexual orientation) inequality. Critically, we know of no study that would have investigated frame use outside Germanic or Romance languages. However, it is conceivable that different linguistic norms (cf. Dover, 2022), grammatical structures, and connotations of terms referring to (dis)advantage could influence frame use.

Finally, the present studies did not investigate the presumed mechanisms behind the interaction of valence and framing. We drew on different cognitive processes involved in (group) comparisons and the processing of positive and negative content to derive our predictions, including basic cognitive processes (e.g., the negativity bias, Kahneman & Tversky, 1979; correspondence principle, Woltin & Epstude, 2023, and feature-positive effect, Newman et al., 1980) and corresponding linguistic practices (e.g., the positivity norm; Brown, 2015; the more-less asymmetry, Hoorens & Bruckmüller, 2015). Future research should experimentally test associated markers such as cognitive fluency, that is, how easily positive versus negative outcomes framed as disadvantage versus advantage are processed (e.g., time to read statements). This would provide insight into the suggested processes and could help tease apart which processes contribute to what extent. Investigating mechanisms could also reveal whether the present findings generalize across illegitimate and legitimate inequalities.

## Conclusion

Confirming our hypotheses, we found a general preference for disadvantage frames of gender and sexual orientation inequality. However, when describing inequality in positive (compared with negative) outcomes, advantage frames were used more. Supporting the validity of these findings, Study 1 used data from real online discourse, while Study 2 replicated these findings in a controlled, experimental study. We conclude that considering how some social groups have more of something positive may be a (more) useful starting point to make privilege and favoring mechanisms visible (than considering advantage in terms of fewer negative outcomes)—which is necessary to fully understand and address inequalities. Practitioners may incorporate these findings in awareness exercises to start reflection and debate on privilege.

## Notes

1. Since data collection, Twitter has restricted research access to the Application Programming Interface (API) to paid, premium accounts.

2. The maximum number of search results per request is restricted by Twitter to 500.

3. The excluded tweets about reverse discrimination also focused more often on the disadvantaged (here: men) than on the advantaged group (here: women), and this was significantly moderated by valence. This suggests that the underlying processes are independent of which group is (allegedly) disadvantaged or advantaged.

4. We did not analyze tweets describing inequality in both negative and positive outcomes because they were very rare in combination with advantage framing, leading to expected cell frequencies below five.

5. In the preregistration, Hypothesis 1 (H1) is worded as follows: “Overall, participants use more disadvantage framing than privilege framing.” Hypothesis 2 (H2) is worded as above, but also uses the term *privilege* instead of *advantage*.

6. Excluding queer participants did not change results. Participants used more disadvantage than advantage frames, *F*(1, 98) = 107.9, *p* < .001, $\eta_{p}^{2}$ = .52, 95% confidence interval (CI) $\eta_{p}^{2}$ = [.39, .62], and the framing by valence interaction was significant, *F*(1, 98) = 264.6, *p* < .001, $\eta_{p}^{2}$ = .73, 95% CI $\eta_{p}^{2}$ = [.64, .79]. Both effects were significant for both domains. See online supplement for descriptive and test statistics, including results of pairwise post hoc tests.
